# A Regulatory Network of Heat Shock Modules-Photosynthesis-Redox Systems in Response to Cold Stress Across a Latitudinal Gradient in Bermudagrass

**DOI:** 10.3389/fpls.2021.751901

**Published:** 2021-11-16

**Authors:** Minghui Chen, Lu Gan, Jingxue Zhang, Yu Shen, Jin Qian, Mengli Han, Chuanjie Zhang, Jibiao Fan, Shengnan Sun, Xuebing Yan

**Affiliations:** College of Animal Science and Technology, Yangzhou University, Yangzhou, China

**Keywords:** bermudagrass, WGCNA, cold stress, heat shock proteins (HSPs), HSFs

## Abstract

Bermudagrass (*Cynodon dactylon* Pers.) is a wild *Poaceae* turfgrass with various genotypes and phenotypes. In this study, 16 wild bermudagrass germplasms were collected from 16 different sites along latitudinal gradients, and different temperature treatments were compiled and used for physiological and transcriptome analysis. To explore the correlation between the key differentially expressed genes and physiological indicators, a total of 14,654 DEGs were integrated from the comparison of different temperature treatments and used for weighted gene co-expression network analysis. Through comparative transcriptome analysis and gene annotation, the results showed that differential gene expression profiles in networks are associated with the plant growth, photosystem, redox system, and transcriptional regulation to cold stress in bermudagrass. In particular, genes encoding HSP70/90 and HsfA3/A8 are not only regulated by temperature stress, but also directly or indirectly interplay with the processes of peroxide scavenging and chlorophyll synthesis under cold stress. Besides, through a weight evaluation analysis of various physiological indexes, we identified an accession of wild bermudagrass with relatively strong cold resistance. These results provide important clues and resources to further study the responses to low-temperature stress in bermudagrass.

## Introduction

Bermudagrass (*Cynodon dactylon*) is a perennial grass of the *Poaceae* family, which is widely distributed in tropical to temperate regions (Taliaferro, 1995). As a warm-season turfgrass, the wild germplasm resources of bermudagrass are abundant and diverse in nature. Additionally, the sensitivity of bermudagrass in different areas to temperature is also variable (Anderson and Taliaferro, 2002). Therefore, wild germplasm resources derived from different latitudes or climatic zones are excellent materials for studying the potential mechanism of their response to temperature stress. For instance, the freezing tolerance of natural accessions is related to some compatible metabolites and transcription factors (TFs) in *Arabidopsis thaliana* (Hannah et al., 2006). Similarly, the phenotypic variation and C*-repeat binding factor* (CBF) gene family were examined in *Populus balsamifera* across different latitudes (Menon et al., 2015).

In plants, the fluidity of the plasma membrane is destroyed and excessive reactive oxygen species (ROS) accumulate under temperature stress, including freezing, chilling, and heat, leading to oxidative stress (Zhu, 2016; Gong et al., 2020). Generally, ROS induction can activate redox-responsive proteins, like protein kinases and TFs that respond to abiotic and biotic stress (Colcombet and Hirt, 2008). Meanwhile, active ROS scavengers can be induced by heat shock protein (HSP), heat shock factor (Hsf), and/or their complexes to protect plants against oxidative damage under heat stress (Driedonks et al., 2015). For example, the *HsfA2* gene of African bermudagrass (*Cynodon transvaalensis* Burtt-Davy) was heat-inducible and exhibited transcriptional regulation of ascorbate peroxidase in *Arabidopsis* (Wang et al., 2016). At low temperatures, cold stress can lead to the inhibition of growth and decreased photosynthesis (Huang et al., 2014). The capacity to reduce photoinhibition and recover photosynthesis during cold stress differs during freezing tolerance in two Zoysiagrass genotypes native to high and low latitudes (Li et al., 2018). Besides, many TFs are involved in cold stress responses and defense, such as CBFs and Hsfs (Hu et al., 2013; An et al., 2020).

Although great progress has been achieved in revealing the response mechanisms of plants to cold stress at various levels, the understanding of the integrated network of regulation is still incomplete. One example is the unique role of HSPs and Hsfs in cold stress response, despite these proteins traditionally being studied concerning heat stress. Furthermore, it is unclear how the HSP-Hsf-Redox-Chlorophyll complexes respond to cold stress and what occurs in the cross talk between different regulatory roles.

To date, transcriptome analysis has provided some molecular perspectives on low-temperature responses in plants, but only in a limited number of species (Chen et al., 2015; Zhang et al., 2018). Our previous study revealed that morphological traits of wild *C. dactylon* displayed significant differences attributed to the influence of native latitude differences (Zhang et al., 2018). These morphological differences are caused by long-term environmental adaptation and natural selection, indicating that the temperature reaction mechanisms in stress response might be different as well. In this study, it is hypothesized that the sensitivity of wild bermudagrass germplasm in response to different temperature stress could be associated with different stress response networks. In this study, a series of RNA-sequencing (RNA-seq) and physiological assays from germplasms collected across 16 latitudes were used to explore the differences between samples upon stress treatments. We integrated differential gene expression profiles to identify critical genes in networks associated with plant growth, photosynthesis, and cold stress response of bermudagrass along a latitudinal gradient. In addition to our transcriptomic analysis, on the basis of weight evaluation analysis of various physiological indexes, our study revealed that one accession of wild bermudagrass has relatively strong cold resistance, which provides valuable materials for future bermudagrass breeding efforts.

## Materials and Methods

### Plant Materials and Treatment

The bermudagrass (*Cynodon dactylon*) materials were collected from 16 different wild sites in different regions of China (Supplementary Table 1). All genotypes were grown in the experimental field of Yangzhou University. In order to eliminate the effect of cytotype on gene expression, three replicated tetraploids with similar growth status were respectively, selected from different latitudes in this study, based on our previous chromosomal observations of the bermudagrass root tip. The 48 samples from different latitudes were transplanted into 48 plastic pots (20 cm depth × 16 cm diameter). The pots were filled with a mixture of sand and organic soil (1:1, v/v). Plants were cultivated in a greenhouse with a photoperiod of 12 h, a temperature of 25°C, and a humidity of 60%. After growth for 1 month, all the samples were equally divided into three groups. Each group was respectively, transferred into their respective phytotron under standardized growth conditions (12 h photoperiod, 20°C). After 7 days, the samples were subjected to 35°C (T35), 20°C (T20), and 5°C (T5) for 24 h. The fully expanded leaves were harvested immediately after the completion of treatment. All of the leaves were frozen in liquid nitrogen and stored at −80°C for RNA extraction.

### RNA Sequencing and Bioinformatic Analysis

RNA-sequencing was performed at Beijing Biomarker Technologies Co. Ltd. (Beijing, China). In brief, total RNA was isolated from mature leaf tissues using TRIzol according to the manufacturer’s protocol. A total of 137 cDNA libraries were constructed as previously described and sequenced by Illumina NovaSeq. After removing the adaptor sequences and low-quality reads, trimmed reads were *de novo* assembled into reference contigs with Trinity software using default settings (Grabherr et al., 2011). Finally, the contigs were clustered into unigenes using the TIGR Gene Indices clustering (TGICL) software system (Pertea et al., 2003). Raw data were submitted to the National Center for Biotechnology Information (NCBI) under the BioProject PRJNA646313 and PRJNA649353.

The transcriptomic resources of *C. dactylon* were submitted as BLASTx queries to functionally annotate expressed genes according to the NCBI non-redundant (Nr) protein sequences, clusters of orthologous groups (COG), gene ontology (GO) database, euKaryotic orthologous groups (KOG), and Kyoto Encyclopedia of Genes and Genomes (KEGG) public databases (Altschul et al., 1997). After predicting the amino acid sequences of unigenes, HMMER v3.1 (hidden Markov models) software was used to compare sequences to the Protein family (Pfam) database to obtain annotation information of unigenes (Finn et al., 2016).

The fragments per kilobase of transcript per million mapped reads (FPKM) were used to represent the expression abundance of each assembled unigene. Differential expression analysis of two samples was performed using edgeR software (Robinson et al., 2010). Fold change (FC ≥ 2) and a false discovery rate (FDR) < 0.01 (*P*-value < 0.05) were used to analyze the significant differentially expressed genes (DEGs) by pairwise comparisons to compare three treatments.

### Trait Measurements and Statistical Analyses

The plant heights (HTs) and root lengths (RLs) were determined by direct measurement and five replications were set in each pot.

Electrical conductivity (EL) assay: a fragment of 0.1 g leaves was soaked in water for 24 h at room temperature, and the initial conductivity (Ci) and final conductivity (C_max_) after autoclaving at 121°C for 30 min were measured with a conductance meter (DDS-11A, Aolilong, China). The relative EL% = (Ci)/C_max_ × 100% (Hu et al., 2016).

The photosynthetic index, including net photosynthetic rate (Pn), stomatal conductance (Gs), and transpiration rate (Ts), were measured by inserting 4–5 individual leaves (second fully expanded leaves from the top) with a portable photosynthesis system (Li-6400, Li-COR Inc., Lincoln, NE, United States). The measurement conditions were: flow rate set at 500 μmol s^–1^ and a fluorescent light intensity of 1,000 μmol m^–2^ s^–1^ (Chater et al., 2018).

Antioxidant assay and malondialdehyde (MDA) content: 0.2 g fresh leaves were ground in a mortar and a crude enzyme solution was extracted with 4 mL of phosphate buffered saline (PBS) buffer. The crude enzyme was applied for the determination of MDA content. After the thiobarbituric acid (TBA) reaction, the absorbance of the supernatant was examined at 532 and 600 nm, and the MDA content was calculated as described in a previous study (Dhindsa, 1981). The peroxidas (POD) mixture contained 50 μL of crude enzyme solution, 1.85 mL sodium acetate-acetic acid buffer (0.1 M, pH 5.0), 0.25 mL 0.25% guaiacol (dissolved in 50% ethanol solution), and 0.1 mL 0.75% (v/v) H_2_O_2_. The reaction system of superoxide dismutase (SOD) mixture contained 1 mL of crude enzyme solution, 2.2 mL sodium phosphate buffer (50 mM, pH 7.8), 0.039 mM methionine, 0.3 nM ethylene diamine tetraacetic acid (EDTA), 0.012 μM riboflavin, and 0.225 μM nitro blue tetrazolium (NBT). The activities of POD and SOD were measured with a spectrophotometer (UH5300, Hitachi, Japan) based on Hu et al. (2012).

Statistical analyses were performed using SPSS21.0 software. For correlation analysis, the Pearson correlation coefficient (*r*) was calculated and a two-tailed test was carried out. For weight evaluation analysis, the technique for order preference by similarity to ideal solution (TOPSIS) method was used in R software.

### Gene Co-expression Network Analysis Between *Cynodon dactylon* and Plant Quantitative Traits

We used weighted gene co-expression network analysis (WGCNA) to build plant traits and bermudagrass gene modules (clusters of genes displaying similarly correlated patterns of transcription) (Langfelder and Horvath, 2008). As it is interesting to understand the relationship between gene expression and plant traits of different bermudagrass germplasms treated at different temperatures, the DEGs of the T5/T20 group and the T35/T20 group of one site were gathered to form a new group using R software, and so on. Finally, all DEGs of the 16 sample sites were gathered to form a differential expression pool. We used the pool of DEGs and quantitative traits to build bermudagrass gene modules. The default WGCNA “step-by-step network construction” analysis was used to build the modules. We calculated the adjacency relationship between genes and constructed a topological matrix. A hierarchical clustering tree with the dissimilarity of the topological matrix was generated, and the modules of dynamic trees were cut by a standard method. Then, similar modules were merged by calculating the eigengenes module. Finally, a cluster dendrogram was formed with a soft-power of 6, a minimum module size of 50 genes, and a distance threshold cut of 0.1 (Supplementary Figure 1).

Additionally, the modules-trait membership was determined by measuring the Pearson correlation between bermudagrass module eigengenes and plant quantitative traits using the default WGCNA relating modules to external information analysis. Then, the correlation and connectivity for each trait to the corresponding correlated module were calculated and shown on the modules-trait membership. According to the significance of the correlation between the trait and gene expression of each module, the mean value of each module represents the significance of the trait within the module, so the module with the largest absolute significance value has the greatest correlation with the given trait. Based on this information, a data set of “hub genes” in the module with the greatest significance of each trait was selected, which was most correlated to the modules of the bermudagrass trait. Finally, the sequences and related information of the “hub genes” dataset were extracted from the transcriptome sequencing data, and then we analyzed and interpreted these genes by Mapman software (Usadel et al., 2009).

### Verification of RNA-Seq Data via Quantitative Real-Time PCR

Total RNAs were extracted as described above for the samples used for RNA-seq. The quantitative real-time (qRT)-PCR analysis was performed to validate the reliability of the RNA-seq data. Four DEGs were chosen for qRT-PCR analysis. Each sample at different latitudes had three replicates. The qRT-PCR assays were performed using the UltraSYBR Mixture (Kwbiotech, Beijing, China) and were conducted on a Roche LightCycler 96 Sequence Detection System. The reference gene, *CdActin*, was used to normalize the expression levels of target genes. All primers are shown in Supplementary Table 2. The expression levels were calculated using the 2^–ΔΔ*Ct*^ method (Livak and Schmittgen, 2001).

## Results

### RNA-Seq Analysis of Bermudagrass in Response to Different Temperatures

In total, 20–42 million clean reads per library were obtained from wild bermudagrass leaves after removing adapter-related and low-quality reads. The GC content ranged from 41.65 to 57.51%, and the respective Q30 percentage (sequencing error rates < 0.1%) was 89.84–94.91%, respectively. After assembly, a total of 553,110 unigenes were obtained with an N50 of 663 bp and a mean length of 510 bp, respectively. Sequence alignment was performed with the clean data of each sample and the assembled unigene library, and mapped reads were used for further analysis. A total of 313,701 unigenes were annotated against the NCBI Nr protein database, Swiss-Prot, KOG, COG, GO databases, and KEGG, respectively.

**FIGURE 1:**
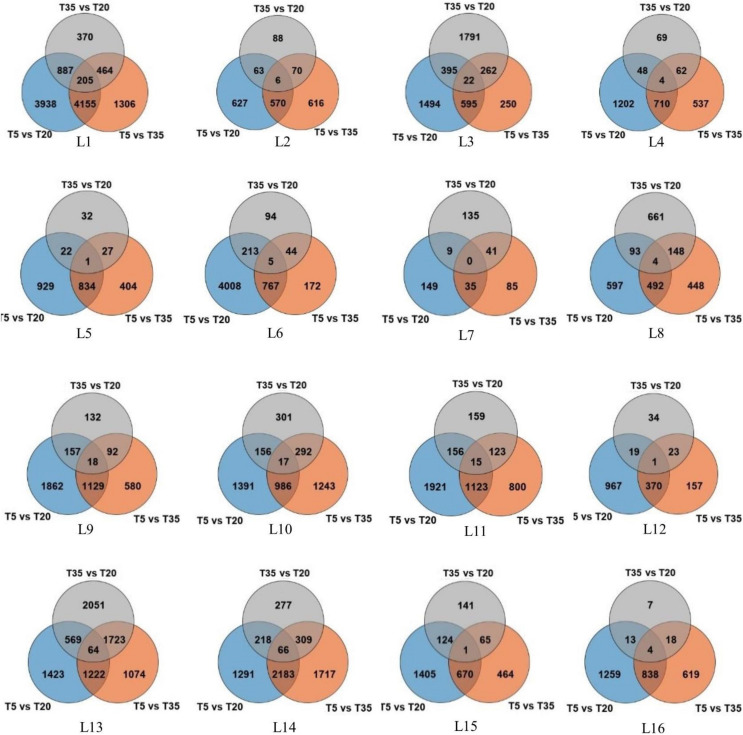
Venn diagram representation of the number of differentially expressed genes (DEGs) identified by RNA-seq analysis in the leaves of bermudagrass from 16 different latitudes across three temperature comparisons.

As shown in Table 1 and Figure 1, the DEGs in the T5/T20 group of most samples along latitudinal gradients are higher than those in the T5/T35 and T35/T20 groups. The results showed that compared with higher temperatures, most genes were expressed following low-temperature treatment. Second, compared to the samples at low and high latitudes, the number of DEGs in the samples at mid-latitude L7 and L8 of each temperature comparison group was less, which suggested that the wild bermudagrass of the two sites may be not sensitive to low or/and high temperature. In addition, among all the same latitudes, the number of DEGs shared by the three temperature comparison groups is less, which indicates that these wild bermudagrasses have altered responses to different temperatures at the transcription level (Figure 1).

**TABLE 1:** The up/downregulated differentially expressed genes (DEGs) in 16 latitudes among three comparisons.

**Comparison**		**T5/T20**			**T5/T35**			**T35/T20**		
**NO.**	**Latitudes**	**Up-regulated DEGs**	**Down-regulated DEGs**	**Total**	**Up-regulated DEGs**	**Down-regulated DEGs**	**Total**	**Up-regulated DEGs**	**Down-regulated DEGs**	**Total**
L1	22°35’40″	3,700	5,485	9,185	2,544	3,586	6,130	953	973	1,926
L2	22°51’48″	279	987	1,266	94	133	227	94	133	227
L3	24°10’31″	803	1,703	2,506	476	653	1,129	1,622	848	2,470
L4	25°05’29″	413	1,551	1,964	443	870	1,313	84	99	183
L5	26°03’49″	295	1,491	1,786	294	972	1,266	29	53	82
L6	27°00’59″	1,550	3,443	4,993	219	769	988	136	220	356
L7	28°09’14″	82	111	193	73	88	161	91	94	185
L8	29°28’32″	324	862	1,186	477	615	1,092	582	324	906
L9	30°25’48″	903	2,263	3,166	577	1,242	1,819	215	184	399
L10	31°18’59″	780	1,770	2,550	1,200	1,338	2,538	435	331	766
L11	32°08’38″	750	2,465	3215	651	1,410	2061	247	206	453
L12	33°09’47″	228	1,129	1,357	167	384	551	43	34	77
L13	34°00’30″	858	2,420	3,278	2,079	2,004	4,083	3,290	1,117	4407
L14	34°54’04″	1,440	2,318	3,758	2,053	2,222	4,275	553	317	870
L15	35°29’26″	574	1,626	2,200	331	869	1,200	158	173	331
L16	36°18’40″	396	1,718	2,114	404	1,075	1,479	16	26	42

In order to better compare the responses of bermudagrass at low and high temperatures, the expression patterns in the T20 group were assigned as the reference samples. A total of 14,654 DEGs from the T5/T20 and T35/T20 comparisons were identified and combined across all latitudes. To understand the function of DEGs in two comparative treatments, these DEGs were searched against the KEGG database using the TBtools software (Chen et al., 2020). Among these pathways, the DEGs of the T5/T20 group were enriched in functions about the ubiquitin system, protein kinases, and glycosyltransferases. Unlike the low-temperature group, chaperone catalysts and metabolism of other amino acids were only found in the T35/T20 comparison (Supplementary Figure 1).

### Weighted Gene Co-expression Network Analysis as a Tool to Decipher the Correlation Between Modules of Bermudagrass Differentially Expressed Genes and Quantitative Traits

**FIGURE 2:**
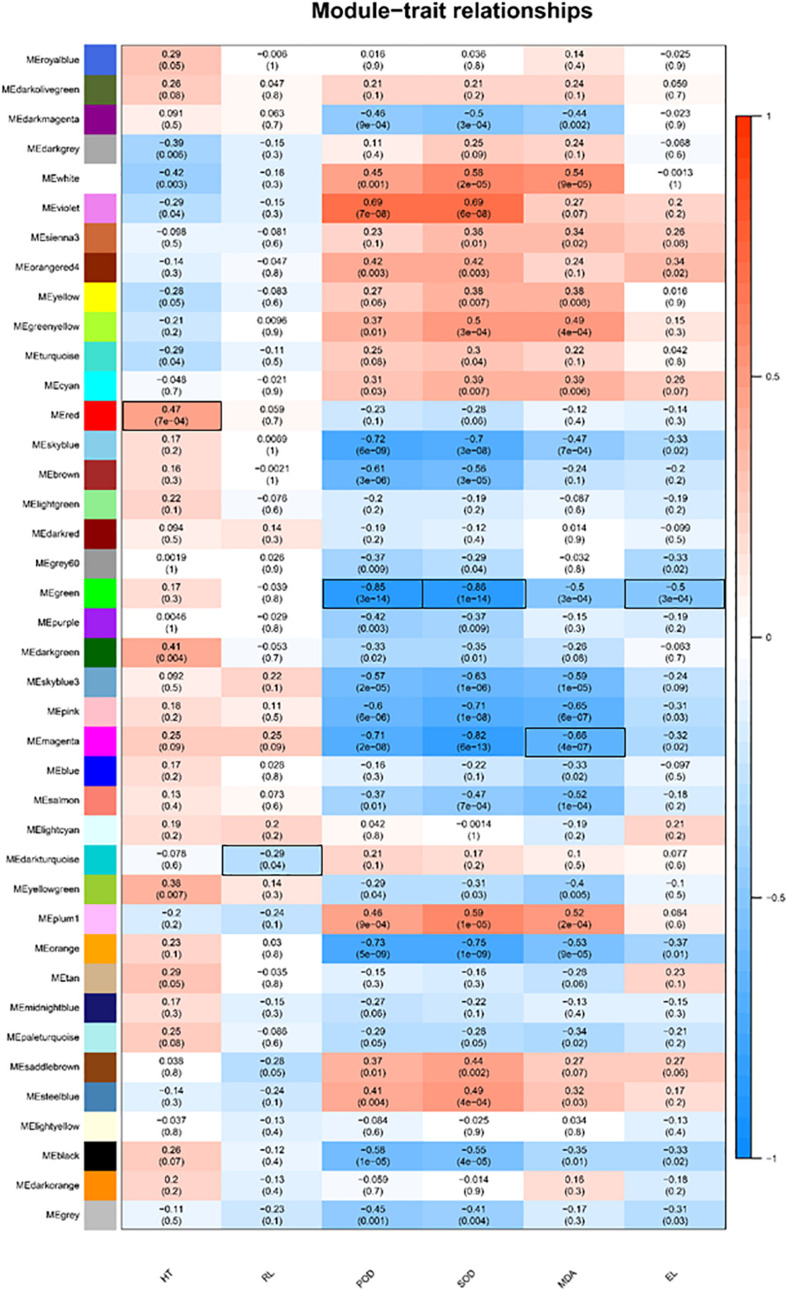
Gene expression modules correlated with quantitative trait of C. dactylon phenotype and physiology, including plant height (HT), root length (RL), peroxidase (POD), superoxide dismutase (SOD), malondialdehyde (MDA), and electrical conductivity (EL). We report the Pearson coefficient and its *p*-value. Highly positive correlations are shown in dark blue and highly negative correlations are shown in dark red. The combination of module-trait marked in black frame will be selected for subsequent annotation analysis.

The WGCNA was used to analyze the association between the 14,654 DEGs identified from T5/T20 and T35/T20 comparisons and physiological indicators. A total of 39 modules related to physiological indicators were observed (Figure 2). Analysis of the module-trait relationships revealed that the “green” module of 256 genes was mostly correlated with SOD (*r* = −0.86, *P* = 1 × 10^–14^), POD activities (*r* = −0.86, *P* = 3 × 10^–14^), and EL (*r* = −0.5, *P* = 3 × 10^–4^). Therefore, the genes in the “green” module may be related to the antioxidant processes of bermudagrass in response to cold/heat stress. The “red” module of 212 genes, “dark turquoise” of 34 genes, and “magenta” of 222 genes appeared to be associated with HT, RL, and MDA content. The genes related to these traits are closely related to the plant growth of bermudagrass under cold/heat stress conditions.

### Effect of Temperature on Plant Heights and Genes Involved in Plant Growth

**FIGURE 3:**
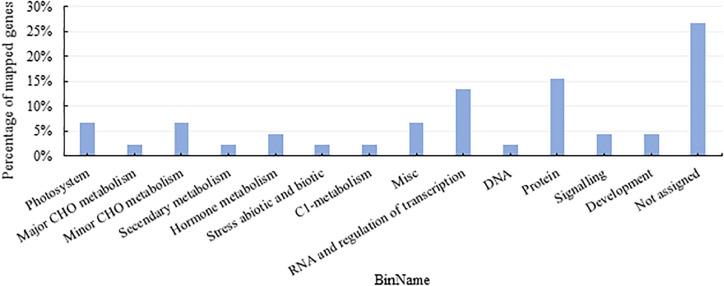
Functional pathway of DEGs from weighted gene co-expression network analysis (WGCNA) modules with close relationship to quantitative traits. CHO, carbohydrates.

First of all, based on the MapMan functional annotation, 724 genes related to different physiological phenotypes in the above modules were identified. As shown in Figure 3, these genes were enriched in the regulation of transcription, protein metabolism, photosystem, minor carbohydrates, and hormone metabolism.

**FIGURE 4:**
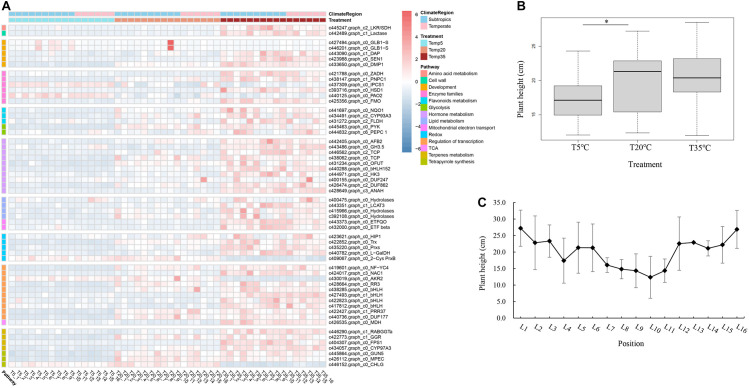
Analysis of key DEGs from related modules with plant growth and development in bermudagrass. **(A)** Heatmap of DEGs from related functional pathways in different bermudagrass samples and treatment groups (T5, T20, and T35). **(B)** Boxplot of plant height of bermudagrass materials from different latitudes after different temperature treatments. The bold black line in the box represents the median, and the asterisk (^∗^) indicates significant difference (*p* < 0.05). **(C)** The plant height of the group of T20 treatment from different positions. The full name or annotation of all gene abbreviations in the study was shown in Supplementary Table 3.

The differentially expressed gene expression profiles in plant growth and metabolism of bermudagrass under different temperature treatment were covered in various pathways, including regulation of transcription, hormone and terpenoid metabolism, tetrapyrrole synthesis, and other processes (Figure 4A). The pathway of transcriptional regulation comprises some TFs, including one NAC (NAM, ATAF1/ATAF2, and CUC2 homologous proteins) domain-containing protein 1 (NAC1), five basic helix-loop-helix DNA-binding superfamily proteins (bHLH), and one pseudo-response regulator 37 (*PRR37*). The homologous genes *PRR37* and *PRR7* were reported to be related to the components of a temperature-sensitive circadian system. The gene expression of *PRR37* was also upregulated in T20 and T35 compared with the T5 group. In addition, some TFs, such as the TCP (Teosinte Branched/Cycloidea/Proliferating cell factors) and DUF (domain of unknown function) family TFs, also participate in the metabolic pathway of plant hormones (Figure 4A).

The primary and secondary metabolites of terpene metabolism, such as phytosterols, chlorophyll, and phytohormones, are involved in regulating plant growth and development. The hormone pathway covers 10 genes, which encode proteins related to biosynthesis and signal transduction of auxin, cytokinin, and ethylene, such as auxin signaling F-box 2 (AFB2), auxin-responsive GH3-like protein 5 (GH3.5), histidine kinase 3 (HK3) of cytokinin receptor, and adenine nucleotide alpha hydrolases-like superfamily protein (ANAH) of ethylene signal transduction. In terms of terpene and chlorophyll biosynthesis, except for *CHLG* encoding chlorophyll synthase, seven genes were covered and upregulated in the T35 treatment group (Figure 4A). Chlorophyll synthase has been shown to perform the esterification of chlorophyllide (a and b), the last step of chlorophyll biosynthesis. Compared to T35 treatment groups, the *CHLG* gene was upregulated in the T5 treatment, while the gene encoding magnesium-protoporphyrin IX monomethyl ester cyclase (*MPEC*) was downregulated in the T5 groups.

Generally, HT is a characteristic of turfgrass growth and a measure of the development and resistance under abiotic stress. Through our analysis of bermudagrass HT at different latitudes after different temperature treatments, compared with the T20 treatment, it is clear that T5 treatment was significantly lower without considering the latitude, while the T35 treatment had no significant difference (Figure 4B). These results were consistent with the expression of the plant growth-related genes as mentioned above.

In order to explore whether latitude affects HT of wild bermudagrass from different regions, the correlation analysis between latitude and HT was performed on the samples of the T20 treatment group (Figure 4C). First of all, the coefficient between latitude and height of bermudagrass was only −0.07 in all regions. Then, according to the latitude data (Supplementary Table 1), the sampling position could be divided into subtropical (L1–L10) and mesothermal zones (L11–L16). So, an exciting discovery is that the height of subtropical bermudagrass was negatively correlated with latitude (coefficient is −0.9120), while the mesotherm zone was positively correlated with latitude (correlation coefficient is 0.8004). Thus, the HT of wild bermudagrass was related to latitude and climate, which also provides a reference range for exploring wild germplasm with cold resistance in these materials.

### Effect of Temperature on Photosynthesis and Expression of Relevant Genes in Bermudagrass

**FIGURE 5:**
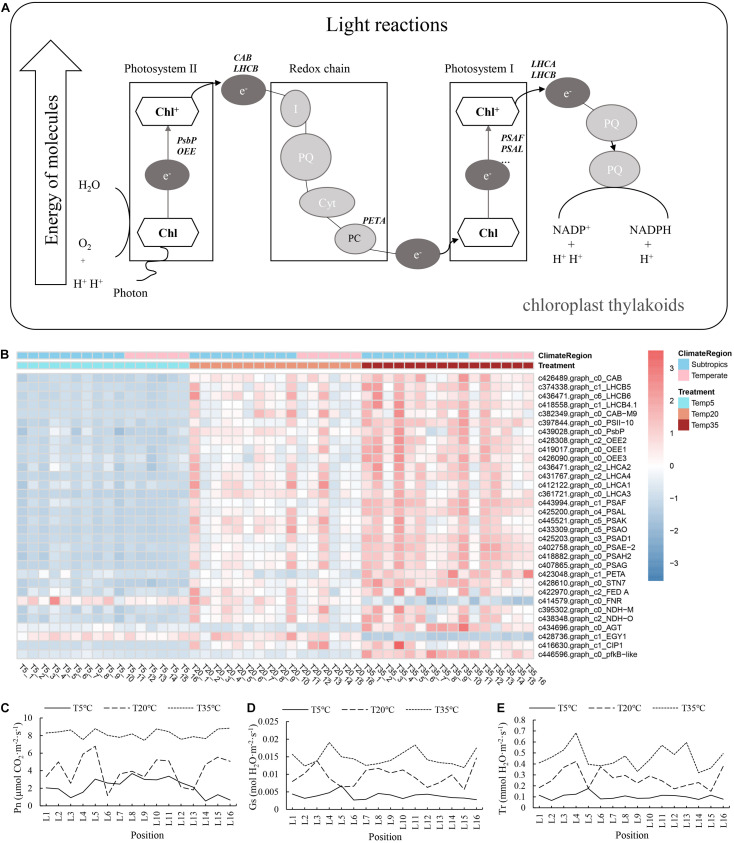
**(A)** Photosynthesis pathways in association with differential expression in bermudagrass. **(B)** Heatmap showing the expression profile of DEGs involved in photosynthesis pathways. **(C–E)** Histogram showing variation in photosynthesis indexes at different temperature, followed by the net photosynthetic rate (Pn), stomatal conductance (Gs), and transpiration rate (Tr).

As mentioned above, the related genes of chlorophyll synthesis in bermudagrass exhibited decreased expression to some extent after temperature stress. We next studied whether the related photosynthetic system was similarly affected. Taking the DEGs in WGCNA modules as experimental objects, the transcriptional expression pattern of genes related to photosynthesis was explored. Analysis of 32 mapped DEGs showed that the most important genes involved in photosynthesis were downregulated under low-temperature treatment, including the genes encoding the subunit of photosystem I (PSA), photosystem I/II light-harvesting complex (LHCA, LHCB), chlorophyll a/b-binding protein (CAB), etc. (Figures 5A,B). Furthermore, the net photosynthetic rate (Pn), stomatal conductance (Gs), and transpiration rate (Tr) of plants with different temperature treatments were determined. The Figures 5C–E showed the effect of the different temperature treatments on the estimated photosynthetic indexes (Pn, Gs, and Tr). The Pn of bermudagrass in the T5 treatment was lower than that of the T35 and T20 groups. Overall, these results indicate that the photosynthesis was blocked at low temperature, while the *CHLG* gene (the enzyme encoding gene of the last step of chlorophyll synthesis) was upregulated in T5 treatment (Figures 4A, 5B).

### Analysis of Differentially Expressed Genes’ Expression Profile and Antioxidase Activity of Bermudagrass in Response to Temperature Stress

**FIGURE 6:**
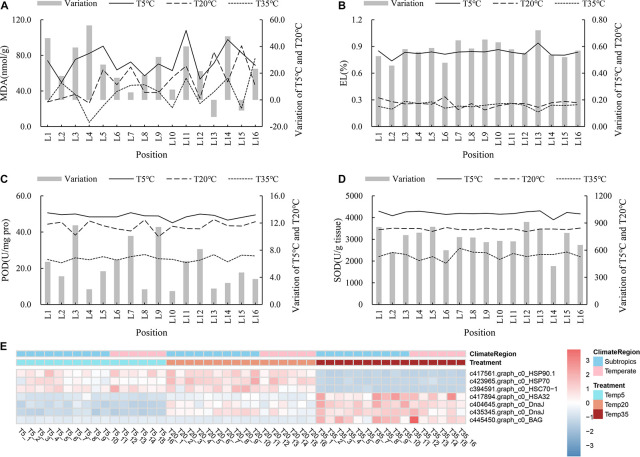
Analysis of stress response to the different temperature in bermudagrass. Malondialdehyde (MDA) content **(A)**, EL **(B)**, POD activity **(C)**, and SOD **(D)** of all bermudagrass materials with T5, T20, and T35 treatment, respectively. The left of histogram showing variation between T5 and T20 treatment. **(E)** Heatmap showing the expression profile of DEGs involved in abiotic stress pathway.

To shed light on the tolerance mechanism of bermudagrass under temperature stress, 11 resistance-related genes obtained from WGCNA association modules were annotated and analyzed. In addition, the MDA content, EL, and antioxidant enzyme activity (POD, SOD, and ascorbate peroxidase (APX)) of the plants with different treatments were also determined. First, Figures 6A,B shows that MDA content and EL of the T5 group were higher than those of the T20 and T35 groups, which indicated that bermudagrass under 5°C treatment suffered damage, but there was no significant difference between the T20 and T35 treatment groups. For the antioxidant system, the activities of POD, SOD, and APX of bermudagrass at most latitudes in T5 treatment were higher than that in the T20 (Figures 6C,D and Supplementary Figure 2). Similarly, the expression levels of some genes encoding POD and SOD were higher in the T5 groups, such as the peroxidase 3 gene (Supplementary Figure 3). With the decrease of temperature, more antioxidant enzymes accumulated, thus delaying the damage caused by temperature stress. In addition, the temperature treatment time in this study was 24 h, which indicated that these wild bermudagrasses responded quickly to low-temperature stress, which laid a foundation for screening cold-resistant germplasm.

Furthermore, plant HSPs and Hsfs are multifunctional, as they regulate protein folding in processes ranging from abiotic stress to plant development. As shown in Figure 6E, the transcript abundance of *HSP90.1*, *HSP70-1*, and heat shock cognate protein 70-1 (*HSC70-1*) were higher in the T5 and T20 treatments compared with T35 groups. On the contrary, heat-stress-associated *32-kD* (*HSA32*), two chaperone *DnaJ-domain* superfamily protein genes, and a member of *Bcl-2-associated athanogene* (*BAG*) family involved in heat stress were upregulated in the T35 treatment.

**FIGURE 7:**
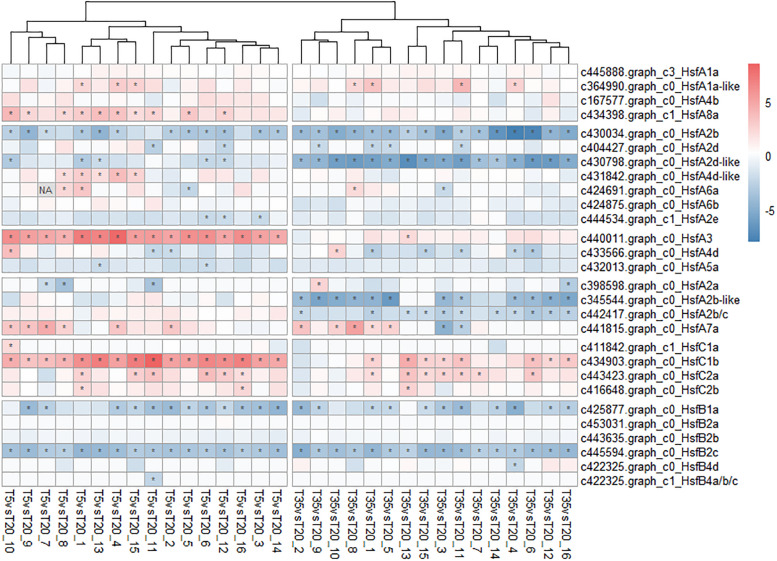
Heatmap of the heat shock transcription factors (Hsfs) genes associated with temperature response in bermudagrass. The abundance (FPKM) of T20 treatment is the reference and the relative expression was determined by the log2 (T5 or T30 _ fragments per kilobase of transcript per million mapped reads [FPKM]/T20_FPKM). The asterisk (*) in the block indicates significant difference (*p* < 0.05) of comparison.

As a critical TF for regulating abiotic stress, 28 HSF transcripts were found from the pool of bermudagrass DEGs and their FPKM values were analyzed. As shown in Figure 7, compared to the expression level of the T20 treatment, *HsfA3, A8a*, and *C1b* were significantly upregulated at 5°C, while *HsfA2d, A2c*, and its homologs were upregulated in the T35 treatment. Besides, *HsfB2c* and B1a were both downregulated at 5 and 35°C, indicating that the expression of both genes was sensitive to temperature. Taken together, these results indicate that a complex regulatory network of the *HSP70*, *HSP90*, *HsfA3*, and *HsfA8a* genes could be more involved in response to low-temperature stress.

### Weight Evaluation Analysis of Cold Resistance in Bermudagrass From Different Latitudes

To explore the cold-resistant germplasms from wild bermudagrass resources collected from 16 regions at different latitudes, the differences of the related physiological indexes between the samples treated at 5 and 20°C were determined, and the weight evaluation of variation was evaluated (we provided Supplementary Table 4 for the detailed data and weight ratio). The physical parameters, including Pn, Gs, Tr, MDA content, EL, POD, SOD, and APX, were integrated by the TOPSIS method for comprehensive resistant evaluation. By calculating the distance ratio between the positive and negative ideal samples, the bermudagrass germplasm resources collected from 16 different areas were sorted. As shown in Table 2, the bermudagrass of the L12 might have strong cold resistance.

**TABLE 2:** Analysis of weight evaluation and rank for the physical index of cold resistance in the bermudagrass collected from 16 latitudes.

**Index**	**Pn**	**Gs**	**Tr**	**EL**	**MDA**	**POD**	**SOD**	**APX**	**Distance_ best**	**Distance_ worst**	**Proximity**	**Rank**
L1	–0.024	–0.012	–0.015	0.023	0.038	0.055	0.018	0.037	0.073	0.120	0.622	3
L2	–0.057	–0.024	–0.039	0.020	0.015	0.037	0.012	0.009	0.124	0.061	0.330	14
L3	–0.031	–0.033	–0.055	0.026	0.032	0.103	0.016	0.011	0.092	0.115	0.557	6
L4	–0.080	–0.014	–0.065	0.025	0.045	0.020	0.017	0.009	0.154	0.070	0.314	15
L5	–0.070	0.001	0.000	0.026	0.021	0.043	0.018	0.009	0.119	0.095	0.443	9
L6	0.026	–0.013	–0.064	0.021	0.014	0.059	0.013	0.008	0.091	0.125	0.580	4
L7	–0.022	–0.028	–0.041	0.029	0.005	0.089	0.016	0.022	0.083	0.110	0.570	5
L8	–0.005	–0.024	–0.041	0.026	0.015	0.020	0.015	–0.014	0.117	0.087	0.426	10
L9	–0.005	–0.021	–0.030	0.029	0.026	0.101	0.014	0.010	0.060	0.130	0.686	2
L10	–0.042	–0.027	–0.044	0.028	0.006	0.017	0.015	0.020	0.129	0.067	0.343	12
L11	–0.032	–0.016	–0.029	0.026	0.033	0.057	0.015	0.010	0.088	0.094	0.518	7
**L12**	**0.010**	**−0.007**	**−0.013**	**0.024**	**0.018**	**0.072**	**0.019**	**0.012**	**0.054**	**0.132**	**0.707**	**1**
L13	0.006	–0.013	–0.023	0.032	–0.010	0.021	0.018	0.016	0.107	0.110	0.506	8
L14	–0.077	–0.022	–0.034	0.024	0.039	0.028	0.009	0.007	0.138	0.070	0.335	13
L15	–0.079	–0.008	–0.008	0.023	–0.006	0.042	0.017	0.000	0.139	0.075	0.350	11
L16	–0.082	–0.040	–0.065	0.025	0.019	0.033	0.014	–0.025	0.165	0.034	0.171	16

*Note: The bold value is the highest ranked bermudagrass.*

### Verification of Gene Expression Through Quantitative Real-Time-PCR

**FIGURE 8:**
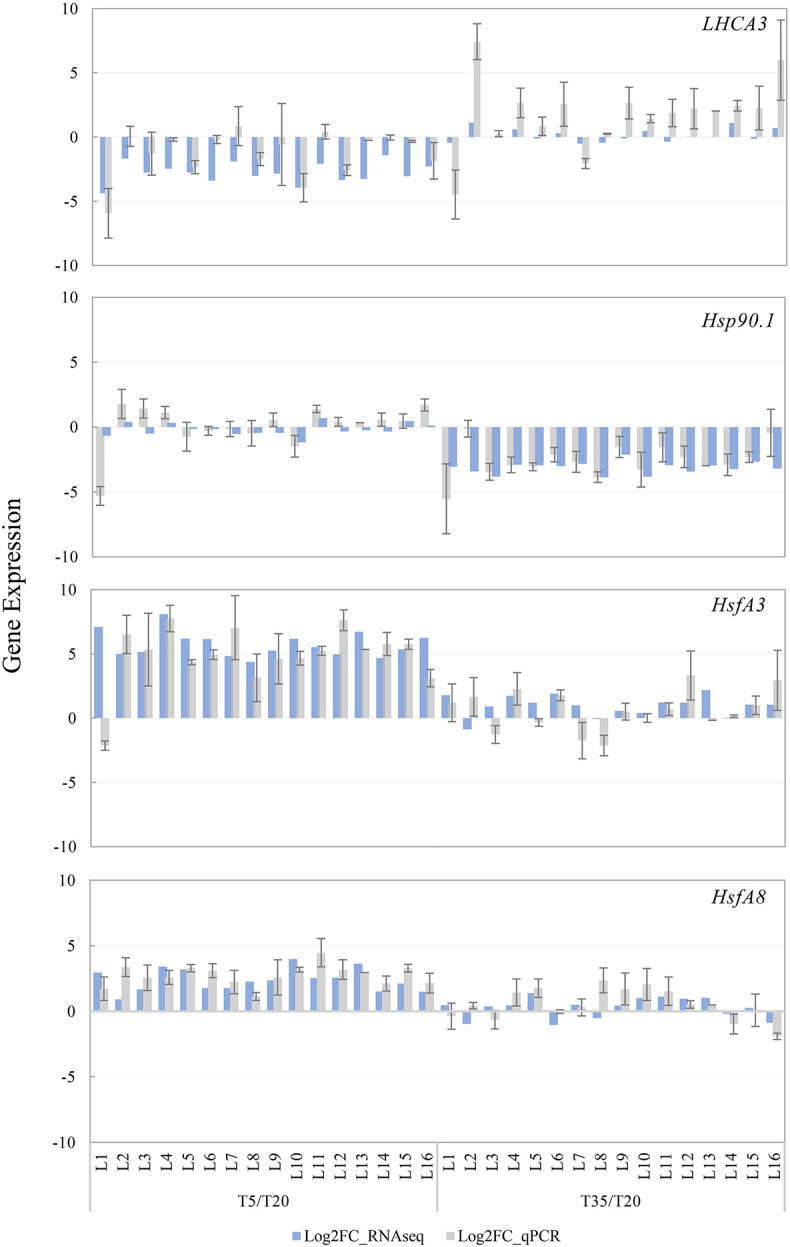
Expression validation of four DEGs through quantitative real-time (qRT)-PCR and RNA-seq analysis. The T20 treatment is the reference and the relative expression was determined by log2 FPKM and Log2 (fold change) compared to the reference. All qRT-PCR reactions were performed from triplicate biological samples. Mean of three replicates ±SE is shown.

In order to further verify the reliability of our transcriptomic data, four DEGs were selected for qRT-PCR analysis, including *HSP90.1*, *HsfA3*, and *HsfA8*, which participate in the response of temperature stress and *LHCA3* involved in the regulation of plant photosynthesis. As shown in Figure 8, the FCs determined by the expression qRT-PCR is consistent with the difference of FPKM in most samples, and their expression patterns were similar. For example, *HSP90.1* was downregulated in the T35 treatment, while *HsfA3* and *HsfA8* were significantly upregulated in the low-temperature group. Taken together, our qRT-PCR analysis indicated that the sequencing results were reliable and provided a basis for subsequent analyses.

## Discussion

Wild germplasm resources of turfgrass are not only valuable resources for selecting excellent cultivars, but also for the mining of novel genes (Yan and Bao, 2014). Bermudagrass is one of the essential warm-season turfgrasses, and its wild resources are widely distributed throughout China from the subtropical to mesothermal zones (Dunne et al., 2019). In our study, bermudagrass materials were collected from 16 wild fields in different latitudes covering subtropical and mesotherm zone. Following the WGCNA of the physiological index and gene expression in all bermudagrass materials treated at different temperatures, an important goal of this study using these wild accessions is to acknowledge the molecular mechanisms relevant for plant growth regulation, photosynthesis, and response to cold stress in bermudagrass, which can be used to identify accessions with improved resistance to cold stress.

### Identification of the Weighted Gene Co-expression Network Analysis Module Highly Associated With Plant Growth and Stress in Bermudagrass

Although the gene expression and physiological performance associated with cold stress in turfgrass have been extensively studied in several wild and commercial species (Shi et al., 2015; Wei et al., 2015; Bi et al., 2016), combined studies of global gene expression, plant growth, photosynthesis, and enzymatic activities in wild bermudagrass from a latitudinal gradient are scant.

Overall, 14,654 DEGs were identified from the combination of T5/T20 and T35/T20 comparisons in all samples from all latitudes, which accounted for the transcriptional regulation of bermudagrass subjected to temperature stress. The 14,654 DEGs enabled us to identify six WGCNA modules of 724 genes, respectively, correlated with HT, RL, MDA content, EL, POD, and SOD activity (Figure 2). WGCNA has been used widely for similar analyses in other studies (El-Sharkawy et al., 2015; Farcuh et al., 2017). Therefore, the identification of some stress-associated “hub genes” was important and meaningful in this study. According to annotation, these hub genes were linked to plant growth and stress response, involved in terpene metabolism, tetrapyrrole synthesis, photosystem, regulation of TFs, and other pathways (Figures 4, 5A, 6B, 7). First, the low temperature has decreased the HT of bermudagrass (Figure 4B), and there are various and complex regulatory networks of related gene expression, such as well-known terpenoid synthesis and hormone metabolism and signal transduction. For the photosynthesis system, it is speculated that cold stress has a negative effect on photosynthesis of bermudagrass from the term of decreased Pn and lower expression of *LHCA* gene and others in electron transport chain under low-temperature treatment. However, the internal relationship between Pn and the efficiency of electron transport chain is worth further exploration. While, for the terms of chlorophyll, the *CHLG* gene expression was upregulated in the low-temperature treatment, which may be due to the adaptation strategy of bermudagrass under cold stress or the negative feedback regulation of the high expression of *MPEC* gene in the chlorophyll biosynthesis pathway.

### The Role of *HSP70* and *HSP90* in Cold Stress

It is known that HSPs are the major induced proteins in response to stress, including cold and oxidative stress (Dumas et al., 2019). The inducible *HSP70s* (70-kDa HSP), as well as the constitutive *HSC70*, act as molecular chaperones, play crucial roles in protecting denatured proteins and prevent substantial damage to plants exposed to cold stress (Franzellitti and Fabbri, 2005). For example, the high expression of *HSP70* transcripts had a strong relationship with resistance to cold stress in *Leguminivora glycinivorella* (Wang et al., 2014), and the mRNA levels of *HSP70s* (including HSP70A, B, C) were increased in *Chlamydomonas reinhardtii* cells exposed to cold stress (Maikova et al., 2016). Similarly, in this study, the expression levels of *HSP70* and *Hsc70-1* genes were upregulated in both 5 and 20°C grown bermudagrass compared with the 35°C treatment (Figure 6E). Although the FC of its expression in the T5 treatment group was lower than that in the T20 treatment group, there was no significant difference. Consistently, there was no significant change in *HSP70* expression of cucumber after 1 day of 4°C treatment compared to a control of 20°C treatment as Ru et al. (2020) reported. These results support that the regulation of *HSP70* and *HSC70-1* is most likely a strategy that bermudagrass uses to react to cold stress. Then, *HSP90* is one of the critical proteins to decrease cellular damage *via* cross talk with other mechanisms in the face of environmental stress (Wang et al., 2004; Hossain et al., 2012). Overexpression of *GmHSP90A* reduced chlorophyll loss and lipid peroxidation levels, so *HSP90A* likely participates in decreasing oxidative stress injury under heat stress (Xu et al., 2013). In the present study, the transcript level of *HSP90.1* and *CHLG* was higher in the T5 and T20 treatments than those in the T35 treatment, and there was no obvious difference in MDA content between the T5/T20 and T35 treatments (Figures 4A, 6A,E).

Genes encoding *HSP70* and *HSP90A*, which are the components of HSP chaperone machine, are both upregulated in *Chlamydomonas reinhardtii* response to cold stress (Maikova et al., 2016). Likewise, *HSP70* and *HSP90* transcripts were also significantly increased in *Ageratina adenophora* after cold treatment, which together could play a fundamental role in protecting cells from stress damage (Gong et al., 2010). In this study, the upregulation of the *HSP70/90.1* gene under low temperature is closely related to the regulation mechanism of bermudagrass adapting to cold stress. And then, the higher expression of the *HSPs* gene in T5 treatment could promote the *CHLG* gene expression and inhibit the MDA content in T5 treatment. Although the net photosynthetic rate is still decreased in T5 treatment, its value was close to that of the T20 groups, which may be due to the mechanism of bermudagrass adapting to cold stress, including *HSPs* regulation. It is suggested that HSPs could direct the protein folding, as well proper assembly of the protein complex such as Rubisco (Park and Seo, 2015). For example, a tomato chloroplast-targeted DnaJ protein could target Rubisco to maintain photosynthesis under temperature stress (Wang et al., 2015). Overall, the accumulation of *HSP70* and *HSP90* genes in bermudagrass to a low temperature of 5°C may reduce the damage under stress by enhancing chlorophyll synthesis and inhibiting peroxidation.

### Heat Shock Transcription Factors in the Regulation of Cold Stress

RNA-sequencing data analysis detected 28 differentially expressed Hsfs among the 14,654 DEGs, but none of them were members of the WGCNA highly correlated modules (Figure 7). However, Hsfs, as the large gene family, are highly redundant and participate in a flexible gene network that regulates abiotic stress responses (Miller and Mittler, 2006). Gad and Ron summarized the findings from a considerable number of studies and described that Hsfs function as hydrogen peroxide sensors in mammals and yeast, and then presented the hypothesis that *HsfA4a* and *HsfA8* might function as potential H_2_O_2_ sensors (Miller and Mittler, 2006). Furthermore, Song et al. (2016) also reported that the expression of *HsfA3* was induced by oxidative damage in *Arabidopsis*. Although Hsf genes in this study are not the “hub genes” of WGCNA, they showed differential expression under different treatments (Figure 7), which indicated that Hsfs might not be directly responsible for the response to cold/heat stress and coincide with flexible and redundant roles. ROS are generally considered as the early signals of various stresses in plants (Mittler, 2004; Karuppanapandian et al., 2011). As mentioned earlier, *HsfA3* and *HsfA8a* of bermudagrass were only upregulated at 5°C (except for HsfA3 in T35/T20_13 samples, Figure 7). Could *HsfA3* and/or *HsfA8a* act as hydrogen peroxide sensors in bermudagrass? Besides, *HsfB2c* and *B1a* were responsive to both 5 and 35°C conditions compared with 20°C, indicating that both of these two genes are sensitive to temperature. Of course, it is necessary to determine whether Hsfs such as *HsfA3*, *HsfA8a*, *HsfB1a*, and *HsfB2c* function as redox sensors in future research.

Under heat/cold stress, hydrogen peroxide can be eliminated by the antioxidative enzymes system, including POD and SOD (Yang et al., 2019). In the current study, the POD, SOD, and APX activities were significantly induced by 5°C stress, and some encoding peroxidase genes were upregulated in T5 and T20 treatments (Figure 6 and Supplementary Figures 1, 2). This begs the question, is there a direct relationship between the levels of antioxidant enzymes, Hsfs, and *HSP70* induced by low temperature? Previous studies have shown that regulation of *HSP70* and SOD-1 were positively correlated, and some induced Hsfs directly regulated antioxidant enzymes in *Arabidopsis*, such as APX (Shin et al., 2013; Song et al., 2016). As well as others, our results demonstrate that POD, SOD, and APX activities and the expression level of *HsfA3/A8a*, *Hsp70/Hsp90* coding genes are both increased in 5°C treated bermudagrass at all latitudes.

**FIGURE 9:**
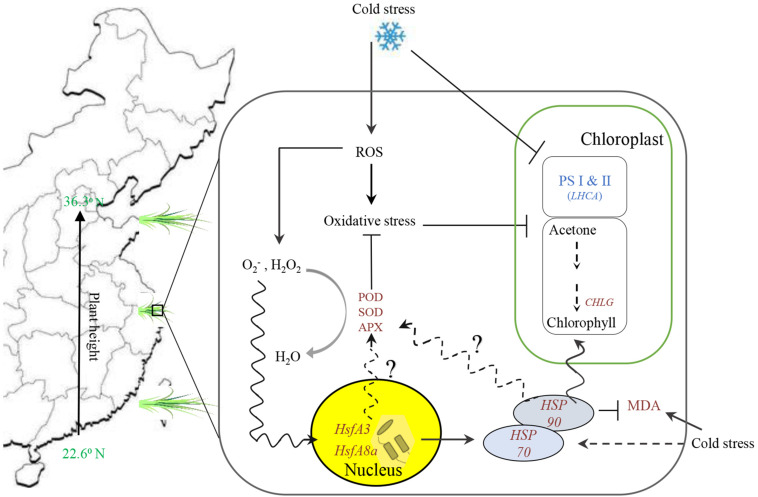
Summary of the overall results count for putative mechanism among heat shock modules (Hsp and hsfs included), photosystem and chlorophyll biosynthesis, and redox system in bermudagrass under cold stress.

Based on differential analysis of physiological performance and gene expression related to plant growth and response to cold stress between 5 and 20°C treated bermudagrass, the above discussion about the regulatory network of heat shock modules-photosynthesis-redox system in bermudagrass is illustrated in a schematic display (Figure 9). Although our results are preliminary, we speculate that there is a direct or indirect interaction among HsfAs, antioxidant enzyme activities, and HSPs to coordinate a systematic response to cold stress.

Finally, based on the weight evaluation analysis of a dataset of the physiological indexes (Pn, Gs, Tr, EL, and MDA content as well as antioxidase activities), there may be an assumption that the germplasm located in Zhumadian (L12) of mesothermal zones with particular climate had good performance under cold stress. Of course, whether this accession could be cultivated as a cold-resistant bermudagrass variety needs further study. At the same time, through comparative transcriptomic analysis of different samples under different temperature treatments, we found that a set of genes encoding HSPs and Hsfs were not only regulated by temperature stress, but also directly or indirectly interplayed with peroxide scavenging and chlorophyll synthesis under cold stress. Of course, the specific functions and regulation mechanisms of the *HSP70/90* and *HsfA3/A8* genes respond to cold stress. This work provides not only essential clues to further clarify how bermudagrass responds to temperature stress, but also provides genetic resources for the cultivation of excellent bermudagrass germplasm.

## Data Availability Statement

The datasets presented in this study can be found in online repositories. The names of the repository/repositories and accession number(s) can be found below: https://dataview. ncbi.nlm.nih.gov/object/PRJNA646313?reviewer=gpoo2sqmv5k m8vc2qp9l8s6cnq, PRJNA646313; https://dataview.ncbi.nlm.nih. gov/object/PRJNA649353?reviewer=ohgrcuab735febammmt4hv jruk, PRJNA649353.

## Author Contributions

XY designed the study. LG performed the data analysis. MC, JZ, YS, JQ, and MH carried out the experiment. LG and MC wrote and revised the manuscript. CZ, JF, and SS made some comments on the manuscript. All authors read and approved the manuscript.

## Conflict of Interest

The authors declare that the research was conducted in the absence of any commercial or financial relationships that could be construed as a potential conflict of interest.

## Publisher’s Note

All claims expressed in this article are solely those of the authors and do not necessarily represent those of their affiliated organizations, or those of the publisher, the editors and the reviewers. Any product that may be evaluated in this article, or claim that may be made by its manufacturer, is not guaranteed or endorsed by the publisher.
