# Supplementary material for: A Regulatory Network of Heat Shock Modules-Photosynthesis-Redox Systems in Response to Cold Stress Across a Latitudinal Gradient in Bermudagrass
Source: Front Plant Sci. 2021 Nov 16;12:751901. doi: 10.3389/fpls.2021.751901 (PMC8636944; doi:10.3389/fpls.2021.751901)
Supplement: Supplementary file 1 [file Data_Sheet_1.docx]

Supplementary Materials

Table S1. The general information of all Cynodon dactylon materials collected from 16 different wild fields in different latitude of China.

| Location_id | Location | Latitude | T5 group | | T20 group | | T35 group | |
| --- | --- | --- | --- | --- | --- | --- | --- | --- |
|  |  |  | Number | Seq-ID | Number | Seq-ID | Number | Seq-ID |
| L1 | Zhong shan | 22°35′40″ | T5_1_1 | T34 | T20-12-1 | T81 | T35-12-1 | T124 |
|  |  |  | T5-1-2 | T35 | T20-12-2 | T82 | T35-12-2 | T125 |
|  |  |  | T5-1-3 | T36 | T20-12-3 | T83 | T35-12-3 | T127 |
| L2 | Guang zhou | 22°51′48″ | T5-5-1 | T13 | T20-5-1 | T56 | T35-5-1 | T106 |
|  |  |  | T5-5-2 | T14 | T20-5-2 | T57 | T35-5-2 | T64 |
|  |  |  | T5-5-3 | T15 | T20-5-3 | T55 | T35-5-3 | T107 |
| L3 | Ying de | 24°10′31″ | T5-10-1 | T28 | T20-10-1 | T75 | T35-10-1 | T118 |
|  |  |  | T5-10-2 | T29 | T20-10-2 | T76 | T35-10-2 | T119 |
|  |  |  | T5-10-3 | T30 | T20-10-3 | T77 | T35-10-3 | T120 |
| L4 | Ren hua | 25°05′29″ | T5-15-1 | T43 | T20-15-1 | T90 | T35-15-1 | T134 |
|  |  |  | T5-15-2 | T44 | T20-15-2 | T91 | T35-15-3 | T135 |
|  |  |  | T5-15-3 | T45 | T20-15-3 | T92 | T35-15-2 | T136 |
| L5 | Gui dong | 26°03′49″ | T5-14-1 | T40 | T20-14-1 | T87 | T35-14-1 | T131 |
|  |  |  | T5-14-2 | T41 | T20-14-2 | T88 | T35-14-2 | T132 |
|  |  |  | T5-14-3 | T42 | T20-14-3 | T89 | T35-14-3 | T133 |
| L6 | You xian | 27°00′59″ | T5-7-1 | T19 | T20-7-1 | T66 | T35-7-1 | T110 |
|  |  |  | T5-7-2 | T20 | T20-7-2 | T67 | T35-7-2 | T111 |
|  |  |  | T5-7-3 | T21 | T20-7-3 | T68 | T35-7-3 | T112 |
| L7 | Liu yang | 28°09′14″ | T5-3-1 | T07 | T20-3-1 | T63 | T35-3-1 | T100 |
|  |  |  | T5-3-2 | T08 | T20-3-2 | T50 | T35-3-2 | T101 |
|  |  |  | T5-3-3 | T09 | T20-3-3 | T51 | T35-3-3 | T102 |
| L8 | Lin xiang | 29°28′32″ | T5-4-1 | T10 | T20-4-1 | T52 | T35-4-1 | T103 |
|  |  |  | T5-4-2 | T11 | T20-4-2 | T53 | T35-4-2 | T104 |
|  |  |  | T5-4-3 | T12 | T20-4-3 | T54 | T35-4-3 | T105 |
| L9 | Xian tao | 30°25′48″ | T5-1-1 | T01 | T20-1-1 | T46 | T35-1-1 | T94 |
|  |  |  | T5-1-2 | T02 | T20-1-2 | T47 | T35-1-2 | T95 |
|  |  |  | T5-1-3 | T03 | T20-1-3 | T48 | T35-1-3 | T96 |
| L10 | Xiao chang | 31°18′59″ | T5-13-1 | T37 | T20-13-1 | T84 | T35-13-1 | T128 |
|  |  |  | T5-13-2 | T38 | T20-13-2 | T85 | T35-13-2 | T129 |
|  |  |  | T5-13-3 | T39 | T20-13-3 |  | T35-13-3 | T130 |
| L11 | Xin yang | 32°08′38″ | T5-8-1 | T22 | T20-8-1 | T69 | T35-8-1 | T113 |
|  |  |  | T5-8-2 | T23 | T20-8-2 | T70 | T35-8-2 | T114 |
|  |  |  | T5-8-3 | T24 | T20-8-3 | T71 | T35-8-3 | T126 |
| L12 | Zhu madian | 33°09′47″ | T5-11-1 | T31 | T20-11-1 | T78 | T35-11-1 | T121 |
|  |  |  | T5-11-2 | T32 | T20-11-2 | T79 | T35-11-2 | T122 |
|  |  |  | T5-11-3 | T33 | T20-11-3 | T80 | T35-11-3 | T123 |
| L13 | Xu chang | 34°00′30″ | T5-16-1 | T137 | T20-16-1 | T93 | T35-16-1 | T138 |
|  |  |  | T5-16-2 |  | T20-16-2 |  | T35-16-2 |  |
|  |  |  | T5-16-3 |  | T20-16-3 |  | T35-16-3 |  |
| L14 | Zheng zhou | 34°54′04″ | T5-2-1 | T04 | T20-2-1 | T61 | T35-2-1 | T97 |
|  |  |  | T5-2-2 | T05 | T20-2-2 | T49 | T35-2-2 | T98 |
|  |  |  | T5-2-3 | T06 | T20-2-3 | T62 | T35-2-3 | T99 |
| L15 | Hui xian | 35°29′26″ | T5-9-1 | T25 | T20-9-1 | T72 | T35-9-1 | T115 |
|  |  |  | T5-9-2 | T26 | T20-9-2 | T73 | T35-9-2 | T116 |
|  |  |  | T5-9-3 | T27 | T20-9-3 | T74 | T35-9-3 | T117 |
| L16 | Ci xian | 36°18′40″ | T5-6-1 | T16 | T20-6-1 | T58 | T35-6-1 | T108 |
|  |  |  | T5-6-2 | T17 | T20-6-2 | T59 | T35-6-2 | T65 |
|  |  |  | T5-6-3 | T18 | T20-6-3 | T60 | T35-6-3 | T109 |

Table S2. Sequences of primers for qPCR in this study.

| GeneName | Primers |
| --- | --- |
| CdActin-F | CCAAGGCAAACAGGGAGAAGA |
| CdActin-R | AGTCGGATGATAGCGTGAGGG |
| Hsfa3-F | GCAGCAACAACGAAATACGAA |
| Hsfa3-R | AACACCAGGATGAATGAACGG |
| Hsp90.2-F | GAGACCGCCCTCCTCACC |
| Hsp90.2-R | CCAGCGTCGTCCTCCAGC |
| LHCA3-F | ACCTCGACGTCAGAACCGA |
| LHCA3-R | CAGCATTGAAACCAGCAGC |
| SEN1-F | ATCCTCGGCTACTTCATCCA |
| SEN1-R | TCAAACGCTTTCTCGTCAG |

Table S3. The list of full name or annotation of all genes in the study.

| **Abbreviation** | **Protein Names** |
| --- | --- |
| 2-Cys PrxB | 2-cysteine peroxiredoxin B |
| AFB2 | Auxin signaling F-box 2 |
| AGT | Alanine: glyoxylate aminotransferase |
| AKR2 | Ankyrin repeat-containing protein 2 |
| ANAH | Adenine nucleotide alpha hydrolases-like superfamily protein |
| BAG | Bcl-2-associated athanogene |
| BBD1 | Bifunctional nuclease in basal defense response 1 |
| bHLH | Basic helix-loop-helix DNA-binding protein |
| bHLH152 | Basic helix-loop-helix DNA-binding superfamily protein 152 |
| CAB | Chlorophyll A-B binding family protein |
| CAB-M9 | Chlorophyll a-b binding protein M9 |
| CHLG | Chlorophyll synthase ChlG |
| CIP1 | COP1-interactive protein 1 |
| CYP93A3 | Cytochrome P450 93A3 |
| CYP97A3 | Cytochrome P450, family 97, subfamily A, polypeptide 3 |
| DAP | Dormancy/auxin associated family protein |
| DMP1 | Dentin matrix acidic phosphoprotein 1 |
| DnaJ | Heat shock protein DnaJ |
| DUF177 | Protein of unknown function DUF177 |
| DUF247 | Protein of unknown function DUF247 |
| DUF862 | Protein of unknown function DUF862 |
| EGY1 | Etheylene-dependent gravitropism-deficient and yellow-green 1 |
| ERD4 | Early-responsive to dehydration stress protein 4 |
| ETF beta | Electron transfer flavoprotein beta |
| ETFQO | Electron-transfer flavoprotein: ubiquinone oxidoreductase |
| EXO70B1 | Exocyst subunit exo70 family protein B1 |
| FED A | Ferredoxin A |
| FLDH | Farnesol dehydrogenase |
| FMO | Flavin-binding monooxygenase family protein |
| FNR | Ferredoxin--NADP reductase |
| FPS1 | Farnesyl diphosphate synthase 1 |
| GGR | Geranylgeranyl reductase |
| GH3.5 | Indole-3-acetic acid- amido synthetase GH3.5 |
| GLB1-S | Globulin-1 S allele precursor |
| GUN5 | Genomes uncoupled 5 |
| HIP1 | HSP70-interacting protein 1 |
| HK3 | Histidine kinase 3 |
| HSA32 | Heat-stress-associated 32 |
| HSC70-1 | heat shock cognate protein 70-1 |
| HSD1 | Hydroxysteroid dehydrogenase 1 |
| HSP70 | Stromal 70 kDa heat shock-related protein |
| HSP81-3 | Heat shock protein 81-3 |
| Hydrolases | Hydrolases superfamily protein |
| IPCS1 | Inositol phosphorylceramide synthase; Arabidopsis Inositol phosphorylceramide synthase 1 |
| Lactase | Beta-galactosidase precursor |
| LCAT3 | Lecithin: cholesterol acyltransferase 3 |
| L-GalDH | L-galactose dehydrogenase |
| LHCA1 | Photosystem I light harvesting complex gene 1 |
| LHCA2 | Photosystem I light harvesting complex gene 2 |
| LHCA3 | Photosystem I light harvesting complex gene 3 |
| LHCA4 | Light-harvesting chlorophyll-protein complex I subunit A4 |
| LHCB4.1 | Light harvesting complex photosystem II 4.1 |
| LHCB5 | Light harvesting complex of photosystem II 5 |
| LHCB6 | Light harvesting complex photosystem II subunit 6 |
| LKR/SDH | Lysine- ketoglutarate reductase/saccharopine dehydrogenase bifunctional enzyme |
| MDH | Malate dehydrogenase |
| MPEC | Magnesium- protoporphyrin IX monomethyl ester [oxidative] cyclase |
| NAC1 | NAC domain containing protein 1 |
| NDH-M | Subunit NDH-M of NAD(P)H: plastoquinone dehydrogenase complex |
| NDH-O | NAD(P)H: plastoquinone dehydrogenase complex subunit O |
| NF-YC4 | Nuclear factor Y, subunit C4 |
| NQO1 | NAD(P)H-dependent oxidoreductase 1 |
| OEE1 | Oxygen-evolving enhancer protein 1 |
| OEE2 | Oxygen-evolving enhancer protein 2 |
| OEE3 | Oxygen-evolving enhancer protein 3-1 |
| OFUT | O-fucosyltransferase family protein |
| PAO2 | Polyamine oxidase 2 |
| PEPC 1 | Phosphoenolpyruvate carboxylase 1 |
| PER | Peroxidase |
| PETA | Photosynthetic electron transfer A |
| pfkB-like | pfkB-like carbohydrate kinase family protein |
| PNPC1 | Cationic peroxidase 1 precursor |
| PRR37 | Pseudo-response regulator 37 |
| Prxs | Peroxiredoxin |
| PSAD1 | Photosystem I subunit D-1 |
| PSAE-2 | Photosystem I subunit E-2 |
| PSAF | Photosystem I subunit F |
| PSAG | Photosystem I reaction center subunit V |
| PSAH2 | Photosystem I subunit H2 |
| PSAK | Photosystem I reaction center subunit psaK |
| PSAL | Photosystem I subunit l |
| PSAO | Photosystem I subunit O |
| PsbP | Photosystem II reaction center PsbP family protein |
| PSII-10 | Photosystem II 10 kDa polypeptide |
| PYK | Pyruvate kinase family protein |
| RABGGTα | RAB geranylgeranyl transferase alpha subunit 1 |
| RLP15 | Receptor like protein 15 |
| RR3 | Response regulator 3 |
| SEN1 | SENESCENCE 1 |
| STN7 | Serine/threonine-protein kinase STN7 |
| TCP | TCP family transcription factor |
| Trx | Thioredoxin superfamily protein |
| WCOR413 | Cold acclimation protein WCOR413 family |
| ZADH | Zinc-binding alcohol dehydrogenase family protein |

Table S4. The detailed information of TOPSIS analysis.

| Variation | Pn | Gs | Tr | EL | MDA | POD | SOD | APX |
| --- | --- | --- | --- | --- | --- | --- | --- | --- |
| L1 | -1.27982 | -0.00358 | -0.06946 | 0.527704 | 46.23997 | 6.27572 | 856.2744 | 15.554 |
| L2 | -3.06655 | -0.00708 | -0.18005 | 0.457363 | 17.92456 | 4.156379 | 575.4809 | 3.9311 |
| L3 | -1.64787 | -0.01 | -0.25343 | 0.580101 | 39.24219 | 11.66667 | 765.5411 | 4.4506 |
| L4 | -4.33449 | -0.00409 | -0.29875 | 0.559508 | 55.77061 | 2.242798 | 792.5463 | 3.5381 |
| L5 | -3.75284 | 0.000317 | -0.00133 | 0.588842 | 26.40724 | 4.897119 | 857.7993 | 3.8081 |
| L6 | 1.414669 | -0.00395 | -0.29272 | 0.478306 | 16.60693 | 6.625514 | 598.6819 | 3.3553 |
| L7 | -1.16191 | -0.00826 | -0.18747 | 0.646075 | 5.68015 | 10.10288 | 744.9549 | 9.1726 |
| L8 | -0.26397 | -0.00716 | -0.18675 | 0.58466 | 18.60386 | 2.222222 | 739.521 | -5.862 |
| L9 | -0.29573 | -0.00625 | -0.13866 | 0.652263 | 32.12152 | 11.41975 | 687.8653 | 3.9606 |
| L10 | -2.28774 | -0.00817 | -0.20101 | 0.631186 | 7.75559 | 1.975309 | 701.8054 | 8.2935 |
| L11 | -1.75172 | -0.00491 | -0.13127 | 0.578997 | 40.03755 | 6.399177 | 697.6834 | 4.0845 |
| L12 | 0.514602 | -0.00195 | -0.06086 | 0.552987 | 21.62826 | 8.148148 | 911.9553 | 4.8192 |
| L13 | 0.309118 | -0.00385 | -0.10507 | 0.721861 | -12.6677 | 2.345679 | 838.2196 | 6.6723 |
| L14 | -4.16578 | -0.00647 | -0.1542 | 0.536726 | 47.63953 | 3.168724 | 423.0468 | 2.7777 |
| L15 | -4.2795 | -0.00246 | -0.03611 | 0.519943 | -7.91261 | 4.711934 | 790.1971 | 0.1404 |
| L16 | -4.41803 | -0.01183 | -0.29928 | 0.568793 | 23.35893 | 3.744856 | 657.632 | -10.19 |
| Weight ratio | 0.198005 | 0.085298 | 0.16204 | 0.102278 | 0.098486 | 0.227952 | 0.061649 | 0.0643 |


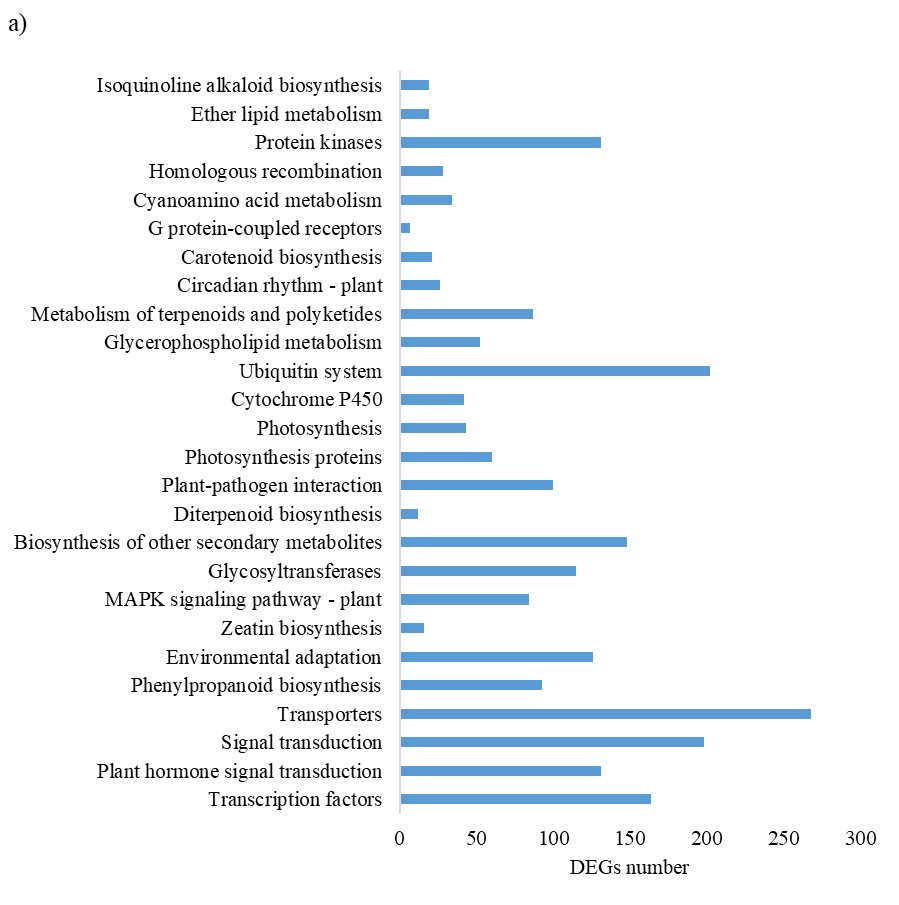

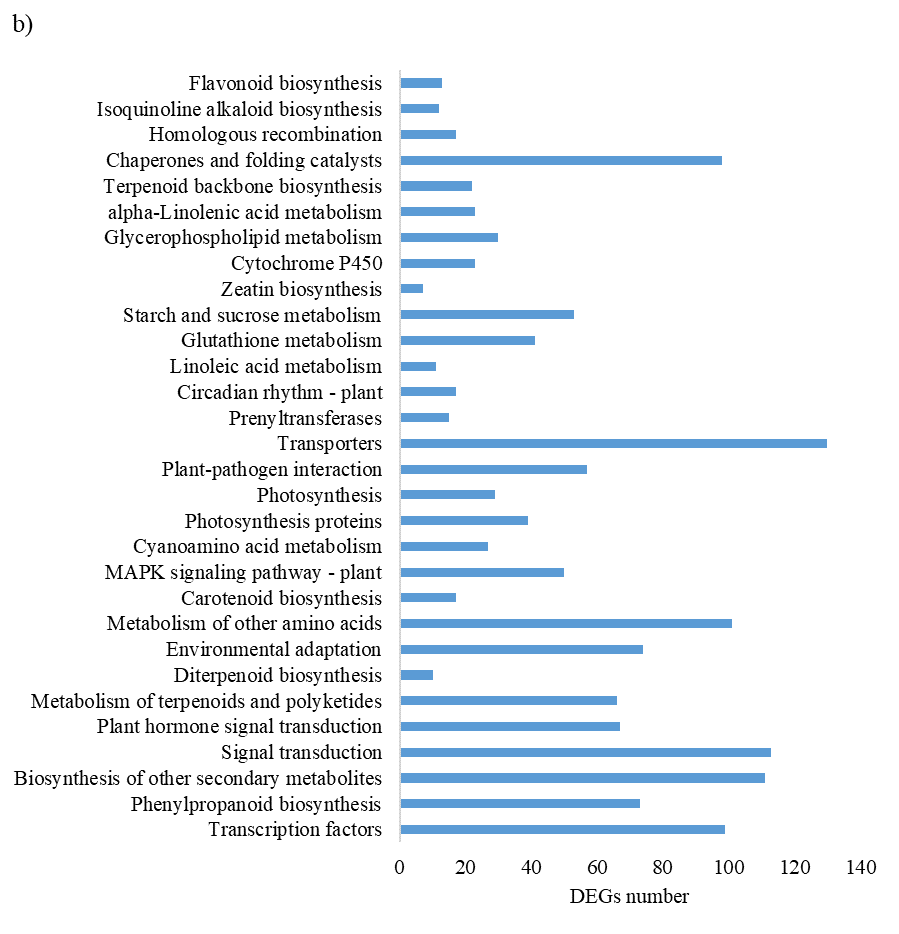


Fig. S1. Number of DEGs enriched in the KEGG pathway based on (a) T5/T20 comparison and (b) T35/T20 comparison.


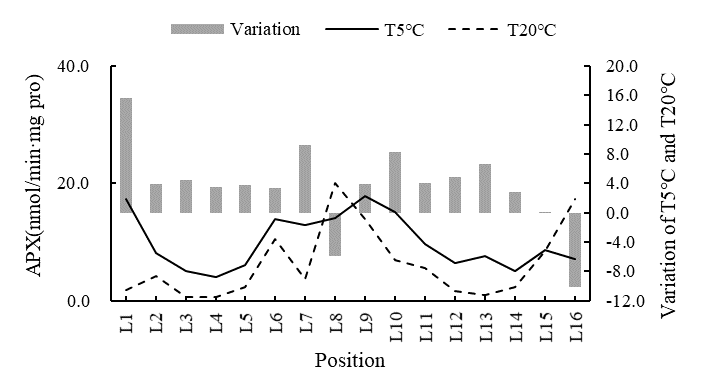


Fig. S2. The APX activity of all bermudagrass materials with T5 and T20 treatment.


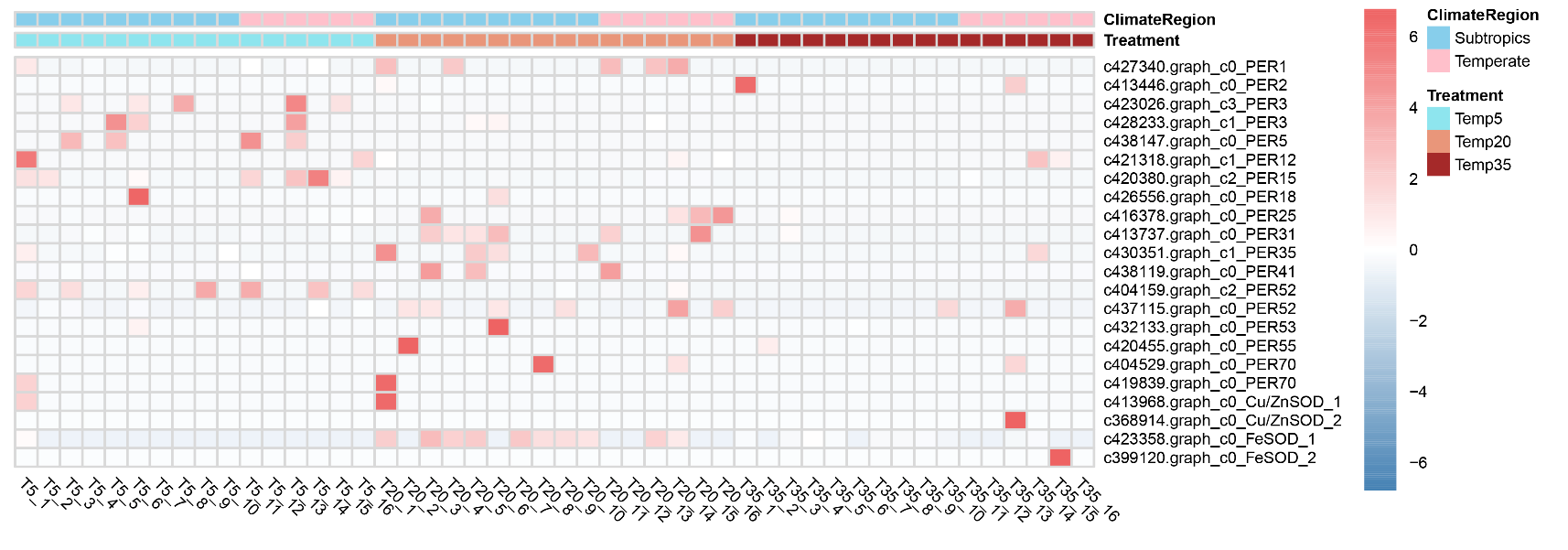


Fig. S3. The gene expression profile of POD and SOD encoded genes in bermudagrass.
